# Utilization of the Winkler scale of plants using big data temperature presented by the Korea Meteorological Administration

**DOI:** 10.3389/fpls.2023.1349606

**Published:** 2024-01-12

**Authors:** Jae-Ryoung Park, Eun-Gyeong Kim, Yoon-Hee Jang, Kyung-Min Kim

**Affiliations:** ^1^ Crop Breeding Division, National Institute of Crop Science, Rural Development Administration, Wanju, Republic of Korea; ^2^ Coastal Agriculture Research Institute, Kyungpook National University, Daegu, Republic of Korea; ^3^ Department of Applied Biosciences, Kyungpook National University, Daegu, Republic of Korea

**Keywords:** Winkler scale, harvest time, grain quality, QTL, breeding

## Abstract

**Introduction:**

Rice is an important food source that can provide a stable supply of calories for most people around the world. However, owing to the recent rapid temperature rise, we are facing social issues related to the increase in the Winkler scale. In this study, a strategy for screening potential candidate genes related to the yield according to the Winkler scale is presented, and the possibility of using a candidate gene identified through sequence haplotype and homology analysis as a breeding source is suggested.

**Methods:**

QTL for the Winkler scale was identified using a population of 120 double haploids derived from a cross between Cheongchoneg, *Indica*, and Nagdong, *Japonica*.

**Results and discussion:**

A total of 79 candidate genes were detected in the identified QTL region, and *OsHAq8* was finally screened. Through haplotype analysis, *OsHAq8* was derived from the *Indica* group and orthologous to Graminae’s activator of Hsp90 ATPase, suggesting that it is a candidate gene involved in yield according to temperature during the growing period. The expression level of *OsHAq8* increased as the Winkler scale increased. The findings of this study can serve as a crucial indicator for predicting harvest time and grain quality while achieving a stable yield through marker selection and adaptation to climate change. Climate change occurs more frequently. In these situations, it is very important to predict harvest time and apply relevant candidate genes to breeding. The candidate genes presented in this study can be effectively applied to rice breeding in preparation for climate change.

## 1 Introduction

Recently, global warming due to greenhouse gases is accelerating, and this phenomenon negatively affects the sustainable development of agriculture (Van Groenigen et al., 2013). The current global average temperature has risen by 0.8°C compared to the early 20th century, and by 2100, the global average surface temperature is predicted to rise by up to 4.8°C (Li et al., 2018; Tollefson, 2020). Owing to the recent rapid rise in temperature, abnormal weather phenomena such as high temperatures, drought, and floods have become unpredictable and have become more frequent and intense (Lesk et al., 2022). This phenomenon is a major cause of yield loss and grain quality deterioration of rice. By 2100, the world’s population is projected to increase to approximately 9 billion, and the Earth’s surface temperature will continue to rise (Davy and Outten, 2020). Accordingly, interest in breeding to maintain the current yield and quality rice cultivars in response to climate change has increased for rice breeders and has become an important breeding goal.

Rice shows different responses to temperature in various growth stages, and a lot of research has been conducted on the effect of temperature on yield loss at each stage (Jagadish et al., 2015; Hussain et al., 2019). In particular, it is the most sensitive to temperature change at the heading date, which is the transition from vegetative growth to reproductive growth, and is a major step in yield loss and grain quality degradation (Arshad et al., 2017; Espe et al., 2017). In addition, the high temperature of the grain filling stage induces increased cellular respiration and oxidative stress, which directly affects grain quality by reducing the flux of carbohydrates in the grain (Deng et al., 2021). In addition, it reduces the supply of assailable products to grain and reduces the grain weight by shortening the grain filling stage due to the increase in the Winkler scale (Arshad et al., 2017).

Rice grain quality is a complex trait that includes various factors and is an important factor in determining price in the global rice market (Chen et al., 2012; Sultana et al., 2022). Grain quality is determined by grain length, grain width, grain thickness, and grain chalkiness (Sultana et al., 2022). High temperature shortens the grain filling stage, reducing the proportion of whole grains and reducing grain size (Tu et al., 2022). By increasing the proportion of grain chalkiness, it affects physicochemical properties such as reduction of amylose content, changes in protein components, and changes in enzyme activity (Yao et al., 2020). High temperature interferes with the biosynthesis of starch, resulting in uneven accumulation of starch, thus resulting in irregular and small-sized starch granules (Yao et al., 2020).

QTL mapping is a technique to identify the most relevant chromosomal regions through the interaction of genes with the environment (Bustos-Korts et al., 2019). In addition, the double haploid population is effective in deriving a mapping population for constructing a genetic map because a large amount of lineage can be grown in a short period of time (Samantaray et al., 2021). In the double haploid population, QTL mapping related to specific traits provides clues to solve the genetic structure of complex traits for quantitative traits. To date, more than 400 QTLs associated with grain size have been reported across 12 chromosomes (Gao et al., 2016; Xu et al., 2020). In addition, QTL related to amylose and protein synthesis affecting grain quality were identified in the recombinant inbred lines (RIL), double haploid (DH), and backcross (BC) populations (Zhong et al., 2011; Zhang et al., 2014; Takemoto-Kuno et al., 2015).

Big data in agriculture include all data related to the cultivation and management of agricultural products, including soil, weather, and management (Delgado et al., 2019). The use of agricultural big data related to image analysis is representative of the use of satellite data (Liu et al., 2019). Satellite data are used to calculate vegetation index based on images and manage cultivation areas. Vegetation index is an index that indicates the state of vegetation through the reflectance of chlorophyll by wavelength of red, near-infrared, and green light (Qiao et al., 2022). Satellite data can be used to estimate crop biomass, death rate, and growth amount in a cultivation area through a combination of spatial information and vegetation index, and the yield of crops can be predicted using cultivation area information (Gašpar et al., 2020). In addition, through monitoring of the occurrence of pests and diseases, it is also used to periodically check the growth status of crops and predict major pests and diseases.

In an era of extreme climate change, the population is increasing. The Winkler scale, yield, and grain quality of the Cheongcheong/Nagdong double haploid population have been investigated for three consecutive years. Through QTL mapping, candidate genes that stably maintained to be less affected by the Winkler scale were screened, and transcription level and haplotype were analyzed. This research method can provide insights into screening candidate genes that can respond to the Winkler scale. It is important to set harvest time to provide sufficient food and maintain grain quality. The Winkler scale can be effective in setting harvest time.

## 2 Materials and methods

### 2.1 Plant material and field management

Cheongcheong (IT228761, IT number is a resource number managed by the National Academy of Agricultural Sciences of Rural Development Administration, Korea) and Nagdong (IT006182), which are cross parents of the double haploid population, were taken and used from the Agricultural Genetic Resources Center of the Rural Development Administration. Cheongcheong (*Oryza sativa* spp. *indica* cv. Cheongcheong) maintains high yield at high temperature, but the grain quality is poor. On the other hand, Nagdong (*Oryza sativa* spp. *japonica* cv. Nagdong) is a sensitive cultivar with low yield in high temperature, but has excellent grain quality. Therefore, these two cultivars were used to derive a mapping population to identify QTL that affect yield and grain quality according to growing season temperature (Lee et al., 2014). The double haploid population, consisting of 120 lines, was obtained from the F_1_ generation through the artificial cross with Cheongcheong and Nagdong. For further studies, Cheongcheong, Nagdong, and 120 Cheongcheong/Nagdong double haploid (CNDH) populations were used for the field test. To investigate the effect of the Winkler scale due to global warming, sowing was conducted at the Kyungpook National University field (36°6′41.54″N, 128°38′26.17″E) on 23 April 2020, 2021, and 2022 (Agricultural Education Center, 39061, 1610, Chisanhyoryeong-ro, Hyoryeong-myeon, Gunwi, Gyeongsangbuk-do, Korea). Thirty days after sowing, transplantation was carried out on the field. The amount of fertilizer applied was N–P_2_O_5_–K_2_O = 9–4.5–5.7 kg/10a, according to the agricultural science and technology survey standards of the Rural Development Administration. In each region, experiments were performed in a randomized complete block design with three repetitions per year. In each block, Cheongcheong, Nagdong, and DH populations were transplanted with 6 rows per line. Each row included 25 plants, and the plant distance was 30 × 15 cm. All field tests were conducted in compliance with international guidelines and laws provided by the Rural Development Administration (RDA) in Korea. In addition, the rice population was cultivated according to local practice. This study complied with the Convention on Trade in Endangered Species of Wild Fauna and Flora (https://www.cites.org/).

### 2.2 Evaluation of investigated traits

The Winkler scale was calculated by adding all the daily mean temperatures from transplanting to the field to harvesting (**Supplementary Figure S1**). Harvesting was carried out 45 days after heading. Growth day was calculated from sowing to 45 days after heading. Near-infrared spectroscopy (NIRS) was used for amylose and protein content.

### 2.3 Construction of the CNDH genetic map

Windows QTL Cartographer software version 2.5 was used for QTL mapping related to Winkler scale, growth day, heading date, yield, amylose content, and protein content (Silva et al., 2012). After analyzing the polymorphism of SSR markers in Cheongcheong and Nagdong, very similar markers were excluded. The selected SSR marker was used to construct a genetic map by Mapmaker version 3.0 (Lander et al., 1987). For linkage mapping, the MAP function of lciMapping software was used (Meng et al., 2015). Also, the marker distance of the genetic map was calculated by the CIM (Composite Interval Mapping) recombination frequency by the Kosambi function. The BIP function of IciMapping version 4.2 software was applied to QTL mapping for Winkler scale, growth day, heading date, yield, amylose content, and protein content (Meng et al., 2015). The LOD threshold for QTL mapping was calculated using 1,000 permutations and an error rate of *p* < 0.05. In addition, to detect QTL, ICIM-ADD (Inclusive Composite Interval Mapping) and ICIM-EP (Epistatic QTL) functions were applied and mapped. To identify additional QTL, a probability of 0.01 and a scanning of 1.0 cM were applied. Finally, QTL with an LOD score of 3.0 or higher was screened to improve the accuracy of the mapped QTL (Zeng, 1993). It was considered significant when the phenotypic variation that could be concurrently explained exceeded 10.0%. The identified QTLs were named according to the method proposed by Mc Couch (McCouch and Jsp, C.H.W.S.N.a.J.R.O.R.O, 2008).

### 2.4 qRT-PCR analysis of candidate genes in the mapping region

Spikes were sampled at intervals of 5 days after heading, and stored at −80°C after cooling with liquid nitrogen until used in the experiment. Total RNA was extracted according to the manual provided by the QIAxcel RNA High Sensitivity Kit (QIAGEN, Cat: 929112, Hilden, Germany). The concentration and quality of the extracted RNA were evaluated using an ultra-microspectrophotometer (ND-2000; Nanodrop, Waltham, Massachusetts, USA). For cDNA synthesis, the qPCRBIO cDNA Synthesis Kit (PCRBIOSYSTEMS, USA), which includes a genomic DNA eraser, was used, and 2 μg of total RNA was used as a template. After that, the manual was followed. An Applied Biosystems™ StepOne™ Real-Time PCR System (Fisher Scientific, Cat: 4376357, Hampton, NH, USA) was used. A 20-μL mix solution contained 10 μL of 2× qRCRBIO SyGreen Blue Mix (PCR BIOSYSFTEMS, Cat: PB20.17-01, Wayne, PA, USA), 1 μL of cDNA, 0.5 μL of forward primer (20 pmol/μL), and reverse primer (20 pmol/μL), and the final volume was adjusted to 20 μL using ddH_2_O. The expression level analysis steps were set as follows: Initially, the sample was held at 95°C for 30 s, followed by 40 cycles consisting of denaturation at 95°C for 5 s and annealing at 60°C for 30 s. Then, there was an additional step of extension at 65°C for 5 s. Finally, there was a final denaturation step at 95°C for 15 s, followed by annealing at 60°C for 30 s and a final denaturation step at 95°C for 15 s. *OsActin*, a housekeeping gene, was used as a control, and the relative expression level of mRNA was calculated by the 2^−ΔΔCT^ method (Rao et al., 2013). For each reaction, at least three repetitions were performed independently. Primer information used for qRT-PCR is listed in the **Supplementary Data** (**Supplementary Table S1**).

### 2.5 Sequence analysis of the candidate gene

A gene information program was used to screen related genes in the QTL region involved in temperature-dependent yield and grain quality. RiceXpro (https://ricexpro.dna.affrc.go.jp/) (Sato et al., 2010), Rice Genome Annotation Project (http://rice.uga.edu/) (Sakai et al., 2013), and Rapdb (https://rapdb.dna.affrc.go.jp/) (Sakai et al., 2013) can obtain information on open reading frames (ORFs) between SSR markers detected by QTL mapping. ORFs allow screening of candidate genes related to traits. The identified ORFs were categorized and screened based on their functions to identify those associated with yield and grain quality in response to temperature variations. For haplotype analysis of candidate gene, SNPs for up/down stream, exon, and intron regions were identified through the Rice SNP-Seek Database (https://snp-seek.irri.org/). Selected candidate genes constructed a phylogenetic tree by applying 1,000-replicate bootstrapping in the Mega X program (https://www.megasoftware.net/) (Kumar et al., 2018). The phylogenetic tree based on DNA sequence was performed after the neighbor-joining method and expressed with the help of the Mega X program (version 7.1). Bootstrap analysis using 1,000 replicate experiments was applied to evaluate the significance of the node. In addition, we used the National Center for Biotechnology Information (NCBI, https://www.ncbi.nlm.nih.gov) and Clustal X version 2.1 (http://www.clustal.org/clustal2/) to identify the rice and Gramineae homology analysis performed on the sequence in the crop. ExPASy (https://www.expasy.org) (Artimo et al., 2012) and Simple Modular Architecture Research Tool (SMART, http://smart.embl-heidelberg.de/) (Schultz et al., 1998) were used to predict the protein interactions of the selected candidate genes.

### 2.6 Statistical analysis

For statistical analysis of the values investigated in this study, R (version 4.1.3, The R Foundation for Statistical Computing) was used. In order to compare and analyze the survey values of agricultural characteristics according to the daily average temperature of each year, a *t*-test and one-way ANOVA were applied for analysis of variance. The significance of the investigated values was analyzed at the *p* < 0.05 level. In addition, to understand the relevance of the investigated characteristics, Pearson’s correlation coefficient was calculated, and the psych package was applied. The corr.test package was applied to compare three or more characteristics. The Corrplot package was applied to visualize various statistics analyzed in this study as graphs and heatmaps.

## 3 Results

### 3.1 Effect of the Winkler scale on yield and grain quality

**Figure 1:**
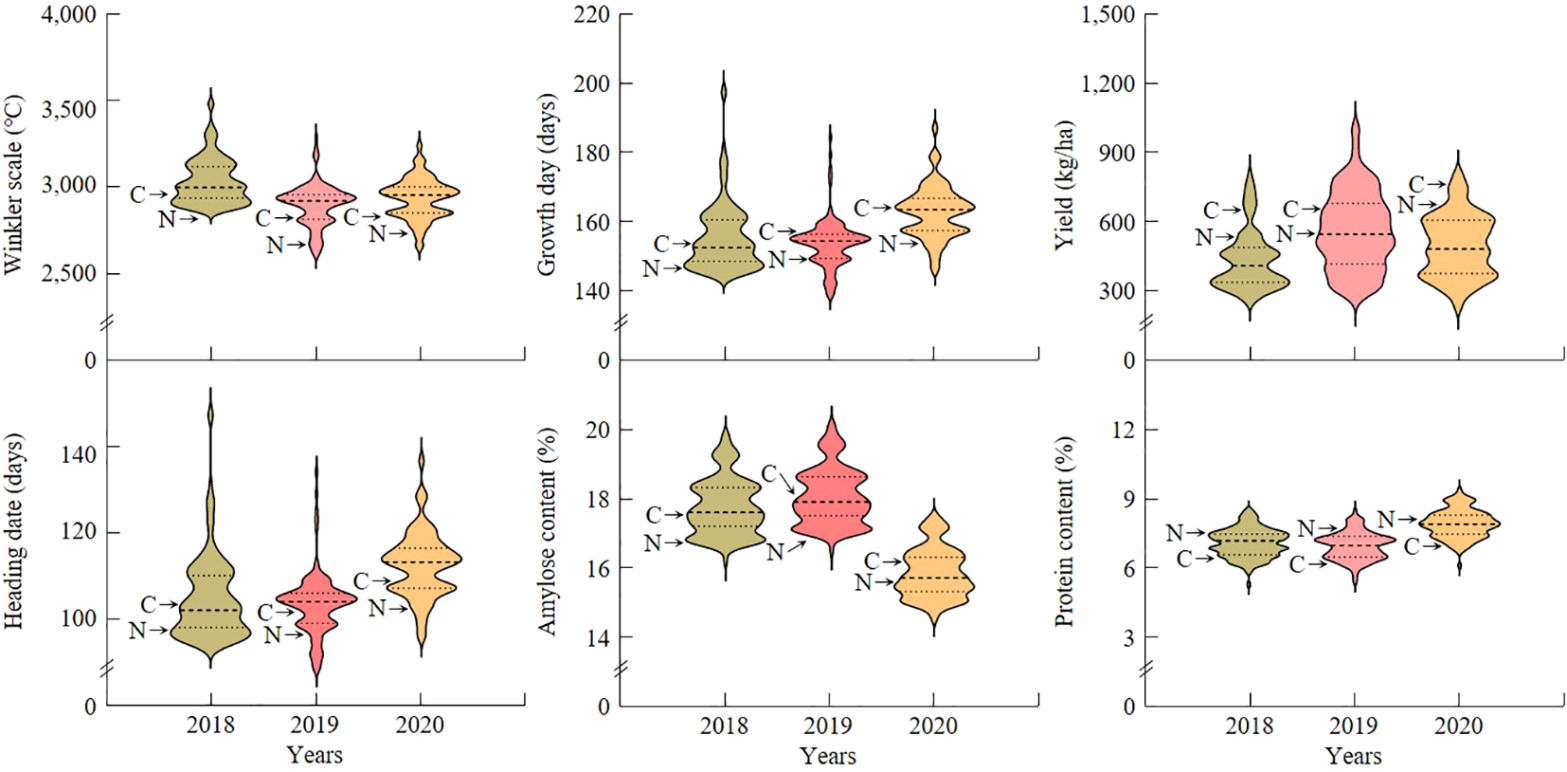
Frequency distribution violin plots of investigated agricultural traits for a population of 120 CNDHs. In 2018, 2019, and 2020, Winkler scale, growth day, heading date, yield, amylose content, and protein content were all normally distributed. This means that all of the investigated traits are quantitative traits that undergo continuous variation. The *X*-axis represents the year investigated and the *Y*-axis represents the range of frequency distribution values of the investigated characteristics. C, Cheongcheong; N, Nagdong.

In order to identify QTL related to yield and grain quality according to temperature changes during the growing season, the Winkler scale, growing days, heading date, yield, amylose content, and protein content were investigated in a population of 120 CNDHs from Cheongcheong and Nagdong. Cheongcheong had a higher Winkler scale during the growing period than Nagdong, and it is a yield (**Figure 1**). Heading date was earlier in Cheongcheong, and growth days were longer in Nagdong. In addition, the content of amylose and protein was higher in Nagdong than in Cheongcheong. The Winkler scale, growth days, heading date, yield, amylose content, and protein content investigated at a population of 120 CNDHs were all continuous and normally distributed, and showed characteristics suitable for QTL mapping. There was no significant difference among the investigated traits in 2018, 2019, and 2020, and the trend of frequency distribution was similar (**Supplementary Figure S2**).

### 3.2 Construction of a genetic map and mapping of QTLs

**Figure 2:**
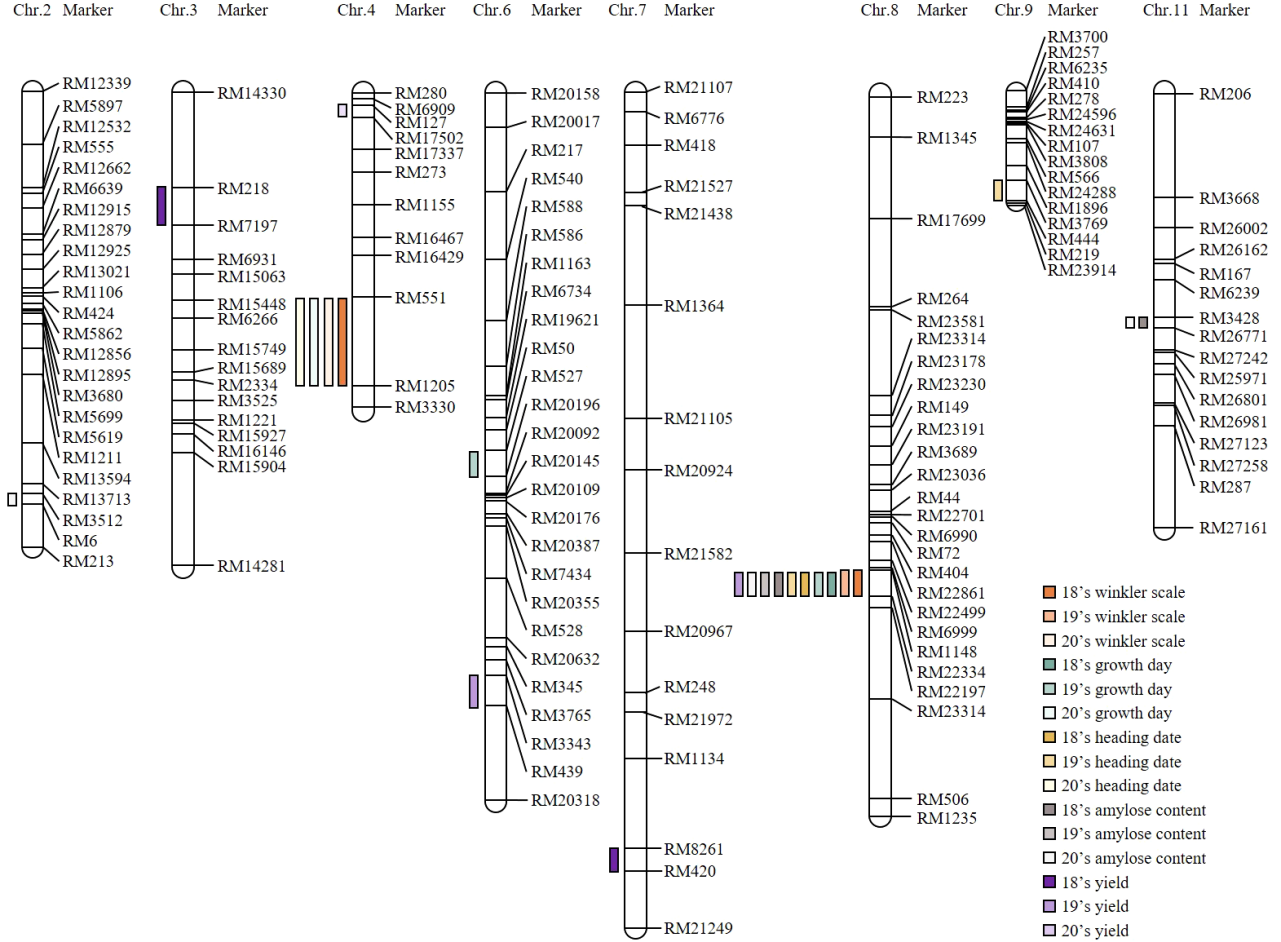
Construct of the 120 CNDH population genetic map and region of the QTL related to investigated agricultural traits. When the investigated values of Winkler scale, growth day, heading date, yield, amylose content, and protein content were applied, the identified QTL was confirmed as a marker region. QTL with a LOD score of 3.0 or higher is marked. RM6999-RM22334 of chromosome 8 is a region that was stably identified for two consecutive years when the investigated values of Winkler scale, growth day, heading date, and amylose content were applied.

A genetic map was constructed for the CNDH population to identify QTL that could affect yield and grain quality according to the Winkler scale. SSR markers with polymorphism were selected through the application of 788 SSR markers to Cheongcheong and Nagdong, and 423 SSR markers were selected out of 288 distinguished Cheongcheong and Nagdong. Of the 423 SSR markers selected, 143 SSR markers had clear PCR bands and were co-dominant markers. Finally, a genetic map for the CNDH population was constructed by applying 143 SSR markers. A total of 143 SSR markers were evenly distributed over 12 chromosomes, and 19–50 SSR markers were distributed per chromosome. The average distance between markers was 10.6 cM, and the total length of the genetic map constructed was 2121.7 cM. QTL mapping was applied by applying the values of Winkler scale, growth days, heading date, yield, amylose content, and protein content, and QTL was detected by applying inclusive composite interval mapping (ICIM) and an empirical threshold with an LOD score of 3.0 or higher (**Figure 2**).

For three consecutive years from 2018, the Winkler scale, growth day, heading date, yield, amylose content, and protein content were investigated, and QTL mapping was performed (**Supplementary Table S2**). As a result, in RM551-RM1205, *qWS4* (LOD, 3.2; PVE, 9.7%), *qWS4-1* (LOD, 3.7; PVE, 11.1%), *qGD4* (LOD, 3.3; PVE, 10.0%), and *qHD4* (LOD, 3.3; PVE, 10.0%) were identified. *qWS4* and *qWS4-1* were identified in the same marker region by the Winkler scale investigated in 2018 and 2020. However, *qGD4* and *qHD4* were identified only in 2020 when the values of the number of days of growth and heading date were applied. In RM1148-RM22334, *qWS8* (LOD, 3.7; PVE, 12.7%) and *qWS8-1* (LOD, 4.2; PVE, 15.1%) were identified in 2018 and 2019. The *qGD8* (LOD, 3.8; PVE, 7.6%), *qGD8-1* (LOD, 3.84.5; PVE, 9.8%), *qHD8* (LOD, 3.1; PVE, 7.6%), *qHD8-1* (LOD, 3.3; PVE, 9.4%), *qAC8* (LOD, 4.5; PVE, 14.1%), *qAC8-1* (LOD, 4.5; PVE, 14.1%), *qAC8-2* (LOD, 3.9; PVE, 12.0%), and *qYD8* (LOD, 3.7; PVE, 8.4%) were identified on RM6999-RM22334 in chromosome 8. In 2018 and 2019, both *qGD8* and *qGD8-1* were identified and both exhibited consistent mapping results over two consecutive years. The *qHD8* and *qHD8-1* were identified in RM6999-RM22334 for two consecutive years applied with heading date. *qAC8*, *qAC8-1*, and *qAC8-2* were QTLs mapped when each amylose content was investigated in 2018, 2019, and 2020. It was detected identically for three consecutive years, indicating that it was very stable and not sensitive to environmental factors. In RM3428-RM27242, *qAC11* (LOD, 3.1; PVE, 9.8%) and *qAC11-2* (LOD, 2.7; PVE, 8.6%) were identified. Additionally, *qAC2* (LOD, 3.4; PVE, 12.0%) was identified. In addition to the QTL mentioned above, QTLs such as *qAC2*, *qGD6*, *qHD9*, *qYD7*, *qYD3*, *qYD6*, and *qYD4* were detected in the rice chromosome, but all of them were detected in different marker regions, or investigated only once in three consecutive years, or the LOD score was investigated lower than 3.0. In particular, when yield was applied, QTLs were identified in different regions for all 3 years, and these inconsistently identified QTLs indicated that they were vulnerable to environmental conditions.

### 3.3 Screening of potential candidate genes in mapped regions

**Figure 3:**
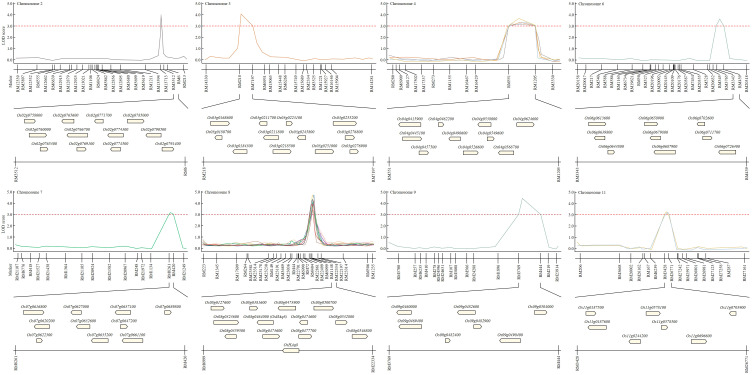
Construct of physical map to screen candidate genes involved in yield and grain quality according to temperature change. RM6999-RM22334 of chromosome 8 is a region mapped by the characteristics of Winkler scale, growth day, heading date, and amylose content for two consecutive years. In this region, 12 potential candidate genes, such as heat sock protein, glycoside hydrolase, and alpha-amylase, may be involved in yield and grain quality according to temperature changes screened.

Candidate genes that can affect the yield according to the Winkler scale were screened in the region where the QTL was identified. A total of 79 candidate genes screened from the identified QTL region were ORFs related to heat shock protein, plant hormone, glycoside transfer, amylose degradation or synthesis, sucrose degradation or synthesis, and cytochrome regulation (**Figure 3**). Among the total candidate genes screened, heat shock protein-related ORFs were identified the most and accounted for 43.0%. ORFs related to plant hormones accounted for 3.8%, ORFs related to glycoside transfer accounted for 22.8%, ORFs related to amylose synthesis or degradation accounted for 12.7%, ORFs related to cytochrome regulation accounted for 8.9%, and ORFs related to sucrose synthesis or degradation accounted for 8.9%. In particular, when candidate genes classified in marker regions were identified in each chromosome, ORFs related to heat shock proteins were screened the most in all QTL regions. Among the QTL mapped regions, candidate genes that could affect grain quality were screened according to the temperature during the growing period in the QTL identified in the same marker region stably for 3 years. We focused on RM6999-RM22334 of chromosome 8, a region mapped identically for 3 years. In the RM6999-RM22334 region of chromosome 8, candidate genes that could affect harvest time and grain quality by the Winkler scale changed during the growing season searched. Candidate genes included proteins related to starch synthesis and degradation, heat shock protein, amylose linking chain, and sugar transporter. **Supplementary Table S3** provides information on candidate genes that were screened in the mapped region.

### 3.4 Genetic distance of candidate genes and association analysis

**Figure 4:**
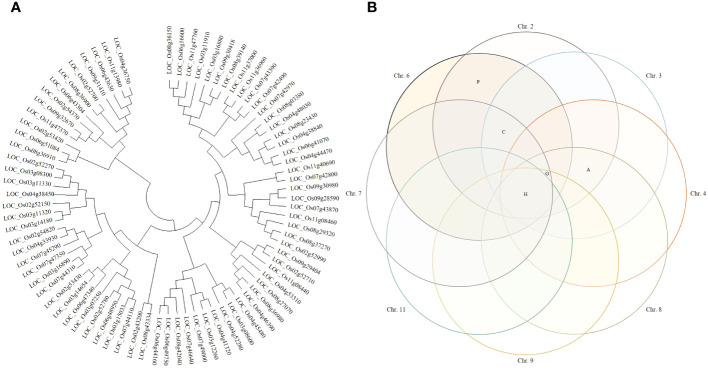
Clustering of potential candidate genes. **(A)** Phylogenetic trees were constructed by genetic distance of candidate genes. Phylogenetic trees were applied with a bootstrap score (≥500) based on 1,000 repetitions. Candidate genes were first divided into 6 groups and further divided into 12 subgroups. Each group was not distinguished by the function of candidate genes. **(B)** Candidate genes were presented in a Venn diagram. In all identified QTL regions, candidate genes related to heat shock proteins were screened. Candidate genes that have a common function in each QTL region were screened and displayed on the Venn diagram. H, heat shock protein; P, plant hormone; G, genes involved in glycoside binding or degradation; A, genes involved in the synthesis and degradation of amylose; C, genes whose expression is regulated by cytochrome.

Candidate genes involved in yield according to the Winkler scale were determined from RM6999-RM22334. A phylogenetic tree was constructed by applying the DNA sequence of the candidate genes, and the candidate genes were distinguished by genetic distance. A phylogenetic tree was generated by applying the neighbor-joining method. For statistical reliability, bootstrap analysis was applied with 1,000 replicate experiments. The results obtained from bootstrap analysis were clear that the phylogenetic relationship was unclear and the bootstrap score was lower in deep nodes. Based on the statistical support of each branch, candidate genes were divided into 6 upper groups and 12 subgroups, which were supported with a bootstrap score of over 90%. Candidate genes were divided into 6 upper groups and each was subdivided into 12 subgroups. When each group was classified by DNA sequence similarity of candidate genes analyzed, it was not distinguished by gene function. In each group, candidate genes with various functions were distributed in various ways. Therefore, each group included candidate genes with functions related to heat shock proteins, plant hormones, glycosides, amylases, and cytochrome (**Figure 4**).

Candidate genes screened in the QTL regions were detected on each chromosome. Candidate genes were classified according to function, and a Venn diagram was constructed to visualize them. The Venn diagram can classify the candidate genes identified in each chromosome according to their common function. Heat shock proteins were identified in all detected QTL regions (chromosomes 2, 3, 4, 6, 7, 8, 9, and 11), and 34 candidate genes were screened. Candidate genes involved in plant hormones were identified on chromosomes 2 and 6, and candidate genes involved in glycoside synthesis and degradation were identified on chromosomes 2, 3, 4, 6, 7, 8, and 9. Candidate genes involved in amylose synthesis and degradation were identified on chromosomes 2, 3, 4, and 8, and candidate genes related to cytochrome activity were identified on chromosomes 2, 3, 6, and 7.

### 3.5 Expression level of candidate genes

**Figure 5:**
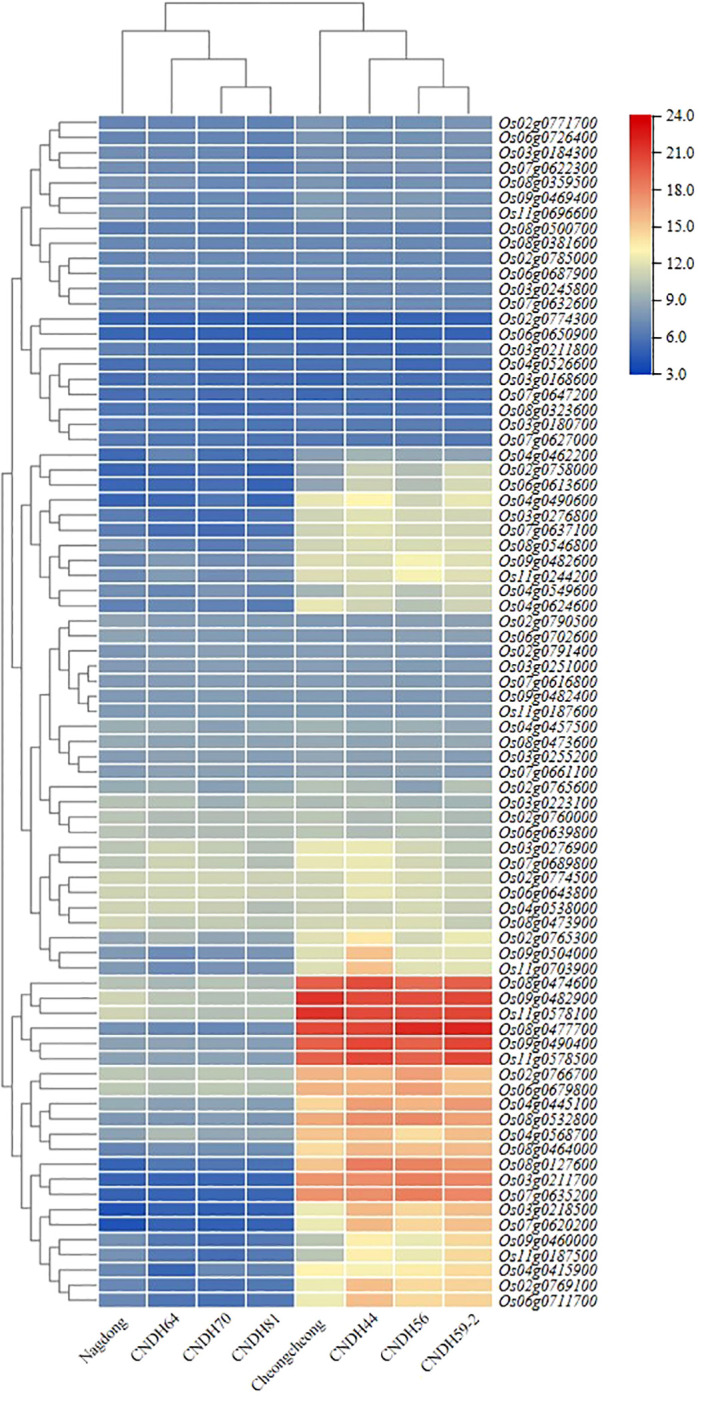
Expression level analysis of potential candidate genes on the heading date. The expression levels of the screened candidate genes analyzed and clustered into 5 groups according to the expression levels. Group 1, group 2, and group 4 had the same level of expression. However, the expression levels of candidate genes belonging to group 3 and group 5 were clearly distinguished in two groups. The expression level of candidate genes belonging to group 5 was strongly maintained.

In the mapping region, candidate genes that can affect rice yield according to the Winkler scale were screened, and their expression levels were compared and analyzed (**Figure 5**). Leaf sampling on the heading date, and *OsActin*, a housekeeping gene, were applied as a control. The expression level of candidate genes was visualized by heatmap, and it was divided into five groups according to the expression level. In addition, the Cheongcheong, CNDH44, CNDH56, and CNDH59-2 group and the Nagdong, CNDH64, CNDH70, and CNDH81 group were distinguished by the expression level of the candidate genes at a high Winkler scale. When the candidate genes were divided into five groups by expression level, the expression level of group 1, group 2, and group 4 was the same in both groups. However, in groups 3 and 5, the population groups were clearly distinguished by the expression level of the candidate genes. In groups 3 and 5, the candidate genes were expressed at high levels in the high yield group, and especially the candidate genes in group 5 were expressed at a very strong level in the Cheongcheong, CNDH44, CNDH56, and CNDH59-2 group. Groups 1, 2, and 4 had the same levels in both groups, but candidate genes belonging to group 4 were expressed at normal levels, and candidate genes belonging to group 2 were expressed at very low levels.

### 3.6 Analysis of gene ontology

**Figure 6:**
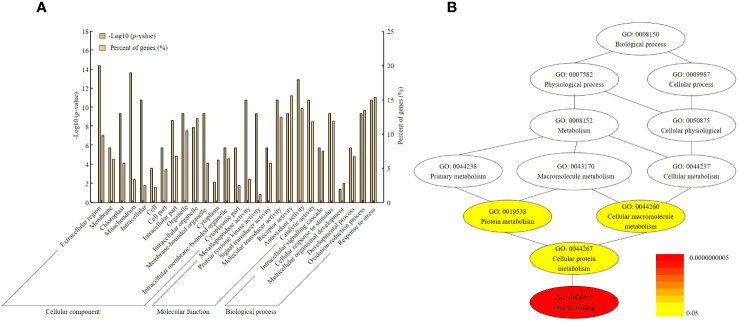
Gene ontology analysis and pathway annotation related to yield determination by Winkler scale. **(A)** GO terms were classified into three categories: cellular component, molecular function, and biological process. **(B)** The pathways of target genes anticipated to exhibit differential expression were categorized. The gradient color of each cluster included in the network represents the *p*-value of each cluster.

Gene ontology (GO) analysis was classified into three categories: cellular component, molecular function, and biological process. Each category was sub-divided into a total of 30 GO terms. Cellular component was classified into 8 GO terms, molecular function was classified into 10 terms, and biological process was classified into 7 terms. In the cellular component, extracellular region, mitochondrion, and intracellular organelle were the top three GO terms. The molecular function was identified to encompass receptor activity, antioxidant activity, and catalytic activity. In biological processes, cellular response to stimulus, oxidation–reduction process, and response to stress were very enriched (**Figure 6**).

In addition, in order to expand the knowledge related to the regulatory network related to *OsHAq8*, the entire gene set of the network including functional annotations was constructed. According to this analysis, the top 12 GOs in the network visualized that their gene products control some vital cellular functions to harvest yield even at a high Winkler scale. Therefore*, OsHAq8* contains a total of 12 genes, with the highest association observed for GO terms such as GO: 0019538 (protein metabolism), GO: 0044260 (cellular macromolecule metabolism), GO: 0044267 (cellular protein metabolism), and GO: 0006457 (protein folding).

### 3.7 Haplotype analysis of *OsHAq8* associated with SNP

**Figure 7:**
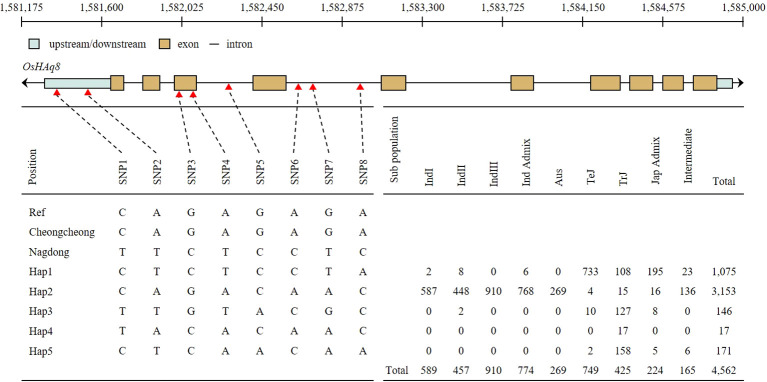
Haplotype analysis of *OsHAq8*. The schematic of *OsHAq8* consists of up/down stream, exon, and intron. *OsHAq8* was classified into five different haplotypes due to eight SNPs. Two SNPs in the upstream, two SNPs in the exon, and four SNPs in the intron were identified. Hap3 accounted for the majority of the subpopulation, and it predominantly distributed within the *Indica* group.

*OsHAq8* haplotype analysis was performed through the Rice SNP-Seek Database. Eight SNP was identified in the upstream, exon, and intron regions of *OsHAq8* (**Figure 7**). Sequence variations were detected in SNP1 (C>T), SNP2 (A>T), SNP3 (G>C), SNP4 (A>T), SNP5 (G>C), SNP6 (A>C), SNP7 (G>T), and SNP8 (A>C), and classified into five haplotypes due to different combinations of SNPs. When referring to the database of the rice genome project, the ratio of Hap2 type was the highest in the sub-population, and identified with a high ratio especially in the Indica group.

### 3.8 Analysis of sequence homology in *OsHAq8*

**Figure 8:**
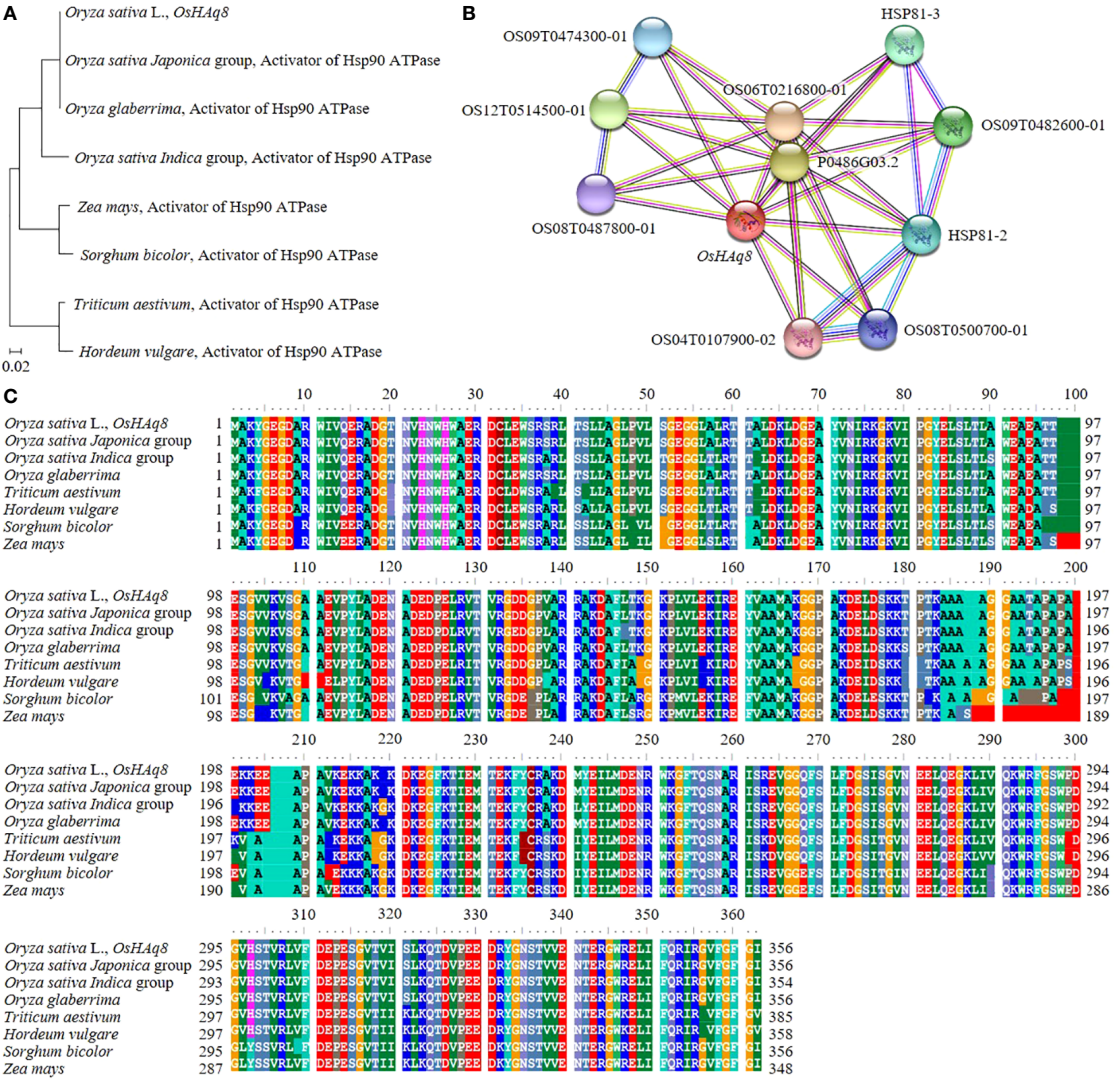
Genetic distance and homology analysis of *OsHAq8*. **(A)** *OsHAq8* is similar to the gramine activator of Hsp90 ATPase, including *Triticum aestivum*, *Hordeum vulgare*, *Sorghum bicolor*, and *Zea mays*, and has a very close genetic distance. **(B)** Also, the homology of the protein sequence of the activator of Hsp90 ATPase is shown. **(C)** *OsHAq8* interacts with transcription factors and proteins involved in hormone signaling, regulating the effect of temperature on yield and grain quality.

In rice, DNA and protein sequences of candidate genes that can affect yield and grain quality according to the Winkler scale were analyzed (**Figure 8A**). For sequence analysis, NCBI’s BLAST function was used. *OsHAq8*, identified in RM22861-RM6999 of chromosome 8, codes for a sequence similar to the activator of Hsp90 ATPase. In addition, *OsHAq8* was identified as very similar to the activator of the Hsp90 ATPase DNA sequence of the *Oryza sativa Japonica* group, *Oryza glaberrima*, the *Oryza sativa Indica* group, *Sorghum bicolor*, *Triticum aestivum*, *Zea mays*, and *Hordeum vulgare*, and their genetic distance was identified with a phylogenetic tree. In particular, *OsHAq8* was analyzed to be most similar to the DNA sequence (identity 100.0%, similarity 99.78%) of the *Oryza sativa Indica* group. In addition, *OsHAq8* was similar and homologous to the activator of Hsp90 ATPase protein sequences of the *Oryza sativa Japonica* group, *Oryza glaberrima*, the *Oryza sativa Indica* group, *Sorghum bicolor*, *Triticum aestivum*, *Zea mays*, and *Hordeum vulgare* (**Figure 8B**). *OsHAq8* interacts with OS09T0474300-01; heat shock protein 90-5, OS12T0514500-01; Hsp90 protein, OS08T0487800-01; Putative heat-shock protein, OS06T0216800-01; Putative cyclophilin-40, OS02T0761100-01; Putative peptidylprolyl isomerase D, OS09T0482400-01; Heat shock protein 81-3, OS09T0482600-01; Heat shock protein 81-3, OS09T0482100-01; Heat shock protein 81-2, OS08T0500700-01; Putative heat shock protein 82, OS04T0107900-02; and heat shock protein 82 in rice and affects yield and grain quality (**Figure 8C**).

## 4 Discussion

Previous reports have indicated that the Winkler scale influences the yield and grain quality of various food crops, including rice (Hatfield et al., 2011; Luo, 2011; Zhu et al., 2022). Global warming is accelerating due to the recent increase in atmospheric carbon and various factors, and the Winkler scale of food crops is rising (Matthews et al., 2009; Wang et al., 2022). As a result, the region and time suitable for growth continue to change, and the Winkler scale rise is negatively affecting yield and grain quality (Krishnan et al., 2011; Wu et al., 2023). In this study, we intend to screen and identify candidate genes that can affect yield and grain quality due to the increase in Winkler scale and apply them to a breeding program in preparation for climate change. Previous genetic analysis revealed that the Winkler scale changes the physiological activity of rice and is directly related to yield [Zhou et al., 2023 (Zhu et al., 2022 #83)]. However, the genes associated with the Winkler scale have not yet been resolved. In this study, we identified QTL related to the Winkler scale and screened candidate genes *OsHAq8*.

In this study, we investigated and evaluated field agricultural traits and grain quality according to the Winkler scale in the CNDH population, derived from the cross between Cheongcheong and Nagdong. The investigated traits were quantitative genetic traits regulated by multiple QTLs and genes. Previously identified QTLs were associated with specific trait regions. In this study, candidate genes were screened in the mapped region, and a strategy for accelerating the breeding of elite rice cultivars in response to climate change was presented through expression and sequence analysis. QTLs associated with temperature-dependent yield and grain quality were identified within the region between RM6999 and RM22334 on chromosome 8. Our results identified QTLs associated with the Winkler scale on chromosomes 2, 3, 4, 6, 7, 8, 9, and 11. Among the identified QTL regions, RM6999 and RM22334 on chromosome 8, which were stably identified for three consecutive years, show that they have a major effect on the Winkler scale.

Compared to previous studies, yield (Zhang et al., 2017)-, grain quality (Yang et al., 2021)-, and stress tolerance (Chen et al., 2021)-related QTL were identified in rice. Xie et al. identified gw8.1 as positively involved in grain width in the RM23201 marker region (Xie et al., 2006). Zhang et al. reliably detected that QSpp8 was involved in spikelets per panicle, QGpp8 was involved in grains per panicle, QHd8 was involved in heading date, and QPh8a was involved in plant height in RM310-RM126 of chromosome 8 (Zhang et al., 2006). The qtl_8.2 and qtl_8.1 mapped by Jagadish were involved in maintaining absolute spikelet fertility at high temperature while being adjacent to the G1073 marker and RIM2A marker, respectively (Jagadish et al., 2010). The QTLs related to yield and tolerance of rice listed above were included in regions similar to the QTLs of RM6999-RM22334 identified from this study in physical locations. However, Lou et al. identified grain size-related QTL on chromosomes 2 and 3 (Lou et al., 2009). In this study, RM6999-RM22334, a QTL region related to grain quality, was detected on chromosome 8, but Xu et al. identified it on chromosomes 1, 2, 3, 5, and 6. In particular, the LOD score of qAAC6 related to amylose content was detected very high at 48.45 (Xu et al., 2015). In this study, QTL related to amylose content was detected, but QTL related to protein content was not identified. The reason why the same characteristics were mapped to different chromosomes and regions in each research is that the materials and environments used in the research are different. Most traits are quantitative traits and are strongly influenced by the environment because they are continuous variables (Consortium, 2003; Zhao et al., 2023). Therefore, it is necessary to focus on regions that are equally stably mapped when studied in various environments and materials (Zhao et al., 2016).

According to the NCBI and RAP-DB databases, candidate genes described as “hypothetical protein,” “expressed protein,” and “retrotransposon protein” were not included, and 12 potential candidate genes were finally identified in RM6999-RM22334 on chromosome 8. The expression levels of the candidate genes were evaluated during the heading date, which is most sensitive to temperature (Arshad et al., 2017; Sun et al., 2022). Heat shock protein improved yield by regulating transcription factors in the environment of heat stress in rice (Al-Whaibi, 2011; Wu et al., 2022b). Amylase and glycoside hydrolase were reported to play an important role in determining grain quality while being involved in the formation of starch (Ye et al., 2019; Yang et al., 2022). Changes in temperature according to rice growth stages can have various effects on yield and grain quality (Hussain et al., 2020). Screening the candidate genes that can play a positive role in maintaining yield and good grain quality according to the changing climate can have a positive effect on breeding accurate and rapid rice cultivars in response to climate change.

GO enrichment analysis aided in gaining a differential understanding of cellular components, molecular functions, and biological processes related to rice yield as per the Winkler scale. GO enrichment analysis suggested that rice controls a very broad and complex reaction approach to maintain yield despite changes in the Winkler scale. In particular, it has the most abundant action in relation to the oxidation–reduction process and response to stress of biological processes. Heat shock proteins prevent protein denaturation, allowing the stable expression and maintenance of gene function when rice is exposed to high temperatures. In this study, the expression level of candidate genes related to heat shock protein increased on the rice heading date.

In order to identify candidate genes in RM6999-RM22334, 12 potential candidate genes encoding proteins were analyzed. Owing to the sequence variation of *OsHAq8*, the expression level was determined differently. In addition, when analyzing genetic distance, *OsHAq8* was found to have close genetic distance and high homology to the activator of Hsp90 ATPase in Graminae. Overexpression of activator of Hsp90 ATPase in *Sorghum bicolor*, *Triticum aestivum*, *Hordeum vulgare*, and *Zea mays* increases the expression of stress-tolerance-related transcription factors, enhances hormone synthesis to strengthen signaling, and ultimately plays a crucial role in controlling the growth and development of crops (Johnson, 2012; Di Donato and Geisler, 2019; Yamada and Watanabe, 2019). From this perspective, the activator of Hsp90 ATPase encoded by *OsHAq8* was utilized in designing a breeding strategy that incorporates functions influencing yield and grain quality in response to temperature.

In this study, candidate genes with the potential to act effectively in maintaining rice yield despite increasing Winkler scale were screened in the mapped regions for three consecutive years. Moreover, they were grouped according to genetic distance. However, each group was not completely classified according to the function of candidate genes. Candidate genes were mostly incapable of distinguishing groups, but there were also candidate genes that could perfectly distinguish the two groups. In particular, candidate genes related to heat shock proteins were all identified in the mapped region. Therefore, it suggested that heat shock proteins play an important role in relation to yield at the Winkler scale. In addition, *OsHAq8* is a gene related to heat shock protein that can distinguish between rice groups by expression level. When plants are exposed to high temperatures, normal protein synthesis is reduced, while the expression of genes such as heat shock protein (HSP) is upregulated, promoting HSP synthesis (Wu et al., 2022a). Some studies have reported that HSP gene expression promotes the activity of protective enzyme genes such as SOD, POD, and CAT (Yurina, 2023). It is proposed that OsHAq8, as an activator of Hsp90, increases the activity of HSP90 at high temperature, assists in the refolding of various proteins, and consequently improves tolerance to stress (**Supplementary Figure S3**).

Crop’s Winkler scale allows the prediction of harvest time and grain quality (Shi et al., 2021). Furthermore, in the face of rapid climate change, maintaining the current yield is equally as crucial as enhancing it. In the future, precise Winkler scale predictions can be made by the Meteorological Administration, and harvest time can be controlled based on the Winkler scale (Long et al., 2022). As a result, there is less potential for a negative impact on the grain quality of the crop. In particular, in rice, the content of amylose and protein changes depending on the temperature of the grain filling stage, which affects grain quality (Lin et al., 2010; Wu et al., 2023). The APEC Climate Center synthesizes weather model results collected from 15 organizations in 11 countries around the world and presents weather forecasts for the next 6 months (Ham et al., 2019). In the future, it anticipated that weather forecasts would become more accurate than they are today, with the ability to provide forecasts for longer periods. This will enable farmers to anticipate harvest times in response to a changing climate and mitigate potential adverse effects on grain quality.

In response to rapid climate change, it must be possible to provide a stable supply of calories to the growing population. Timely and reliable yield forecasts are essential and a prerequisite for preventing climate risks and ensuring food security, especially due to climate change and increasing extreme weather events. Yield and grain quality are affected by harvest time, and the Winkler scale can help predict harvest time (Zhang et al., 2021). Research on yield and protein content analysis are conducted by analyzing chlorophyll content using an image analysis device (Wang et al., 2014; Wan et al., 2020). In addition, the yield and grain quality are analyzed by examining the RGB value of the leaves, and are used to predict yield (Qiu et al., 2021). These methods have become more precise, and the expected analysis values and actual survey values have become very close.

Global temperatures are presently on the rise, and the global population is also growing. The Winkler scale of rice can forecast through the utilization of diverse weather and environmental data. Based on this information, rice yield, harvesting time, and grain quality may be influenced. Here, we suggest that *OsHAq8* is a potential candidate gene that can be involved in rice yield and grain quality depending on temperature. In addition, RM6999-RM22334, for which *OsHAq8* was identified, contains various potential candidate genes that regulate yield and grain quality. However, the effect of potential candidate genes of RM6999-RM22334, including *OsHAq8*, on physiological changes and molecular biological functions of rice according to temperature is unclear. However, to fully understand the function of *OsHAq8*, additional research is needed to develop transgenic plants or genome-editing plants using CRISPR/Cas9. Increasing the cumulative temperature of rice decreased yield and had a negative effect on grain quality. Applying *OsHAq8*, which is related to the Winkler scale identified in this study, to the breeding strategy suggests the possibility of improving temperature-related tolerance. Therefore, it is necessary to elucidate the molecular mechanism by additional studies. Overall, the findings of this study hold practical significance as they can serve as breeding materials to facilitate the achievement of breeding objectives aimed at enhancing both yield and grain quality in response to temperature variations.

## 5 Conclusions

The corresponding QTL was located at RM6999-RM22334 on chromosome 8, and 12 potential candidate genes including *OsHAq8* were also identified. *OsHAq8* was finally selected by qRT-PCR and sequence variation analysis, and it was confirmed to be a homologous Graminae’s activator of Hsp90 ATPase. Since 1975, the global average temperature has risen by 0.15–0.20°C every 12 years, and it is predicted that the global temperature will rise by more than 3.5–8.0°C by 2100. This study presents a rapid and efficient breeding strategy for the identification of QTLs and candidate genes associated with yield and grain quality in relation to temperature variations during the growing season. Through additional studies, we will verify the molecular mechanism function of *OsHAq8* by breeding transformation lines and genome-editing lines.

## Data availability statement

The original contributions presented in the study are included in the article/**Supplementary files**, further inquiries can be directed to the corresponding author/s.

## Author contributions

JP: Conceptualization, Writing – original draft, Writing – review & editing. EK: Data curation, Investigation, Writing – review & editing. YJ: Formal analysis, Writing – review & editing. KK: Supervision, Writing – review & editing.
