# Supplementary material for: Utilization of the Winkler scale of plants using big data temperature presented by the Korea Meteorological Administration
Source: Front Plant Sci. 2024 Jan 12;14:1349606. doi: 10.3389/fpls.2023.1349606 (PMC10811219; doi:10.3389/fpls.2023.1349606)
Supplement: Supplementary file 1 [file DataSheet_1.docx]

**Appendix A. Supporting information**

**Supplementary Table S1.** qRT-PCR primer set for analyzing expression levels of candidate genes.

| Primer name | Forward/Reverse | Sequence (5’ to 3’) |
| --- | --- | --- |
| *OsActin* | Forward | ACCACAGGTATTGTGTTGGACTC |
|  | Reverse | AGAGCATATCCTTCATAGATGGG |
| *Os02g0758000* | Forward | GACCAGATCAAGGCGGAGAT |
|  | Reverse | GCTGCTCCTCCTTCACCTT |
| *Os02g0760000* | Forward | CTTCATCTCCCTCCTCTGCA |
|  | Reverse | TTCCGACGCATCATATCCCA |
| *Os02g0765300* | Forward | AGCCGGACTATGACCAGAAC |
|  | Reverse | CTTGGTGGTGAAGTCGAACG |
| *Os02g0765600* | Forward | AGGGCTACGCATACATCCTC |
|  | Reverse | CTCCTTGAGACCCCAATCGA |
| *Os02g0766700* | Forward | GACATGATGTGCTTCGGTGG |
|  | Reverse | AACTCCTCCAGCGTGATCTC |
| *Os02g0769100* | Forward | CGGGAAGGTGAAGAAAGGGT |
|  | Reverse | TAGAGGTAGGCGATCGGGAT |
| *Os02g0771700* | Forward | CGTGTACCTCTTCGCTCTCT |
|  | Reverse | CTTCTGCTGGTTCGGGTAGA |
| *Os02g0774300* | Forward | TCAGAAGGGAGAGCGACTTG |
|  | Reverse | TCCTCAAAGCGTCTCCCAAT |
| *Os02g0774500* | Forward | ATGATGCTCAATGTCGTCGC |
|  | Reverse | CTGAATAGTTGCCAGCCCAC |
| *Os02g0785000* | Forward | GGGAGAGGGAAGAACAGGTT |
|  | Reverse | CGATGGTGTCCGGGATAAAC |
| *Os02g0790500* | Forward | CATTACCTTCTGCCGCTCAC |
|  | Reverse | GGAAAGAATGGTAGAGCGCG |
| *Os02g0791400* | Forward | CTGTACAGTGCCGTGACAAG |
|  | Reverse | AGCCAACCGAAACCAAAGAC |
| *Os03g0168600* | Forward | CCCAGTAGCAGGAATCCACA |
|  | Reverse | TGATCTTGGCCCTTTCTGGT |
| *Os03g0180700* | Forward | GGTTGAAACTGAGGCGTGTT |
|  | Reverse | CCGATCCAAGCAAAAGAGGG |
| *Os03g0184300* | Forward | TCCTCGCCTTCTTCTTCCTC |
|  | Reverse | CGGTCGAGGAGGTAGGAGTA |
| *Os03g0211700* | Forward | GGATACAACGTCACCCTCCA |
|  | Reverse | TTGTGCTTATGAAGGCTGGC |
| *Os03g0211800* | Forward | TTGCCTGTCTCTGCGTCTTA |
|  | Reverse | TAACAGTTCAGGTGGTGGCA |
| *Os03g0218500* | Forward | GCTACTGTGATGCTGCTGTG |
|  | Reverse | TGGCGCATGACATTCTGAAG |
| *Os03g0223100* | Forward | CGTGTTCTTGGTGAGTGTGG |
|  | Reverse | ATACCGCGCCTTCTCATGAG |
| *Os03g0245800* | Forward | GTCATGGAGGACGACAAGGA |
|  | Reverse | CACCATCACCTTCACCTCCT |
| *Os03g0251000* | Forward | AACACCTTCACCCACCGTT |
|  | Reverse | CACTAATGCACGCGTTGAGA |
| *Os03g0255200* | Forward | GACTTGTCTCTTCGTTGGCC |
|  | Reverse | TCGCGGTGCATAACAGAAAG |
| *Os03g0276800* | Forward | CACCATCAAGAGCGACAAGG |
|  | Reverse | CTCATCCTCGGCCTTGTACT |
| *Os03g0276900* | Forward | CTGGTGGATGGTGCTTAGGT |
|  | Reverse | CGGCCTCAGAACAAAAGACC |
| *Os04g0415900* | Forward | GGCCATCACCATCCCCAA |
|  | Reverse | TACGTCGTCATCAGCCCG |
| *Os04g0445100* | Forward | GACCAGATCAAGGGCCCG |
|  | Reverse | GATGCTCTTCTTGCCACCG |
| *Os04g0457500* | Forward | TTGCAGGTCTGTATGAGGCA |
|  | Reverse | GTGACACTCTGAATGCACGG |
| *Os04g0462200* | Forward | GAAGACAAGCAGCCAGACG |
|  | Reverse | AAACCAAGTGCGACAGGAAC |
| *Os04g0490600* | Forward | CGAGGGCATATGACAAGCAC |
|  | Reverse | CAACCAAAATGCAGTCCCCT |
| *Os04g0526600* | Forward | CAAGCTGGTGTCGTGCAG |
|  | Reverse | CCTTCTTGAAGACCACGACG |
| *Os04g0538000* | Forward | GGATTTTGAGACGGCGATCC |
|  | Reverse | CATCTCAAGGTAAACGGCGG |
| *Os04g0549600* | Forward | CTGAGGCATTGTGTGGGTTC |
|  | Reverse | GCCTTGTGTTGACCAGGTTT |
| *Os04g0568700* | Forward | AGCAACTTCTCCTCCTTCGT |
|  | Reverse | CTTCTCCCCTCTCCTGAAGC |
| *Os04g0612700* | Forward | AAGCCTCTCCAGATCCCAAC |
|  | Reverse | AGTCGATCATCTCCTGCGTT |
| *Os04g0624600* | Forward | ACAAAGCAGGGGATAGGGTC |
|  | Reverse | CGCCATGCATCCAAATCTCA |
| *Os06g0613600* | Forward | CCAGAGACAACATCAAGGCG |
|  | Reverse | GGCGGGTTGTTGATTAGCTC |
| *Os06g0639800* | Forward | GCAACAACTTCGAGCTCCTT |
|  | Reverse | AAGTGGAAAAGCAGACTGGC |
| *Os06g0643800* | Forward | AAGAACACGAGGCTGGAGAA |
|  | Reverse | GGCTTCCTCCTTCTCCAACT |
| *Os06g0650900* | Forward | CCATTTCCAGCGGATCAACG |
|  | Reverse | CAGTACTCCAGCCTCTCCTC |
| *Os06g0679800* | Forward | AGATTCGATGCGGACCTAGG |
|  | Reverse | GCCTTGACATTCCCAGTGAC |
| *Os06g0687900* | Forward | GAGTGGGCTGGTTTTGTGTT |
|  | Reverse | GATTTCCTCGCCGTTCTCAC |
| *Os06g0702600* | Forward | GCAGCTGTCAAACTCCCAAA |
|  | Reverse | TGGCAATGGTGTGTTCTGTG |
| *Os06g0711700* | Forward | CTAAGCGCCCCTACTGCAA |
|  | Reverse | GAGCCGCTTGTGATTCTGG |
| *Os06g0726400* | Forward | GTTGTCCATAGCCATGCGAG |
|  | Reverse | TGGTAGCCCCTATCTCCTGT |
| *Os07g0616800* | Forward | TTCGTGCGCACAACTACAAA |
|  | Reverse | CTCAATGCACCTTGGAGAGC |
| *Os07g0620200* | Forward | GTCGGGCCATCTAGAGGTAG |
|  | Reverse | CCACTTTCTACGCCTCCAGA |
| *Os07g0622300* | Forward | GTTGTGCCAGTTTATCCGGG |
|  | Reverse | GGCGACTGTAGTGACTTTGTC |
| *Os07g0627000* | Forward | GGCTTGAAGATGCTGCACTT |
|  | Reverse | GTCTTCCAAGGCCCTGAGAT |
| *Os07g0632600* | Forward | AACTACGGAGGATGGAGCAC |
|  | Reverse | GCCAAACAGTGACGTGCTAA |
| *Os07g0635200* | Forward | ATCAAGATCCGGCTCGCTAA |
|  | Reverse | CGCACGTCTCCAACATCAAT |
| *Os07g0637100* | Forward | AGCCGAGTCAAAATTCAAGCA |
|  | Reverse | TCGGAACACCAGACCTCATC |
| *Os07g0647200* | Forward | TTGATTTGGTTTGGGCGGAC |
|  | Reverse | CGTACCGGTCGAAGTGATCC |
| *Os07g0661100* | Forward | TGGTCTTGAAGTTGGGCAGA |
|  | Reverse | TTGCGCTTCAATGTCCGTAG |
| *Os07g0669700* | Forward | GGGTGGTGTTCATTGCATGT |
|  | Reverse | TACAACAATGGGCGCAAACA |
| *Os07g0689800* | Forward | CACCTCAAGCTCCTCTACGA |
|  | Reverse | CGATCTCACTGAAGCGAACG |
| *Os08g0127600* | Forward | CAAGGAAGCGGACGAGAATG |
|  | Reverse | CTGCATCATCACAGTGTCGT |
| *Os08g0323600* | Forward | GAAAGGTCCAACGCTGAGTC |
|  | Reverse | ACTTTGTTACCGCGGGAAAC |
| *Os08g0359500* | Forward | GGGCGGAATGGGTGATATGA |
|  | Reverse | CATCATCTCAGCCATGCCAC |
| *Os08g0381600* | Forward | ACTCCATGTTCTTAAGGGGCT |
|  | Reverse | AGGTGAAGGTGCTGGTCTG |
| *Os08g0464000* | Forward | GCAGTTCAGCCTCTTCGATG |
|  | Reverse | CACGATCAGCTTCCCTTCCT |
| *Os08g0473600* | Forward | AGCCCAAGTTCTCTTCCAGG |
|  | Reverse | CAGAAAGTTGTACCACCCGC |
| *Os08g0473900* | Forward | TGAAGAACACCAGCAGCTTG |
|  | Reverse | TGTGCTTGACCCGAGTTACA |
| *Os08g0474600* | Forward | TCATCTGTTGAGCATGGGGA |
|  | Reverse | TGGATTTGGGATCACGTAGGA |
| *Os08g0477700* | Forward | GACCCTACTCCCTGCCATTG |
|  | Reverse | CATCACCGGAACCAACTGC |
| *Os08g0500700* | Forward | TGGAGATCAACCCGGAGAAC |
|  | Reverse | GGACTTGTCGTTCTTGTCGG |
| *Os08g0532800* | Forward | TTCAAGGTTCAGGCGGGG |
|  | Reverse | TGGATGAGAAGACGGCAGAA |
| *Os08g0546800* | Forward | CGTTCCTGACCAAGACGTAC |
|  | Reverse | TCGTCGTTCCAGGAGATGAC |
| *Os09g0460000* | Forward | GTGTCTTCCAGTGAGCGAAA |
|  | Reverse | TGTCGGGAAGATTGCAGTTG |
| *Os09g0469400* | Forward | AGCTTGAGACACTGGGTTGA |
|  | Reverse | GGTGCATCAAGAGGACAACC |
| *Os09g0482400* | Forward | AGCGCCATTTGACCTCTTTG |
|  | Reverse | GTCCATGATAAACACCCGGC |
| *Os09g0482600* | Forward | AGCGCCATTTGACCTCTTTG |
|  | Reverse | GTCCATGATAAACACCCGGC |
| *Os09g0482900* | Forward | GGAGGAGGAGAAGAAGGCAG |
|  | Reverse | TCACGTGAGAAGCCGACAT |
| *Os09g0490400* | Forward | CGTTCATGGACTGCTTCGAG |
|  | Reverse | AGGCGGTCGATGTAGATGAG |
| *Os09g0504000* | Forward | GTCTACAACCTCGGCAACAC |
|  | Reverse | CTTCTTGCCGAGGAGCTTCT |
| *Os11g0187500* | Forward | GAGCAAGAACAACATCGCCA |
|  | Reverse | TGGTCCTGTTGTAGCTCCTG |
| *Os11g0187600* | Forward | GGTTGATATGCTTCTCCGCG |
|  | Reverse | GGATTGCAGTGTTCCTTGGG |
| *Os11g0244200* | Forward | GTGCTCACCGTCACCGTG |
|  | Reverse | CTTCACCACCTCCGCCTC |
| *Os11g0578100* | Forward | CGAGATCATTGAGTGCACGG |
|  | Reverse | TCCAGAACTCCACTTTGCCT |
| *Os11g0578500* | Forward | AGCCGCATTCAGGTTTGTTT |
|  | Reverse | CCTCCTTGGCCACTTCTCTT |
| *Os11g0622800* | Forward | ACCTGCTGACTACCCTTTGT |
|  | Reverse | TGACTTCCCTGGTTGGTTCA |
| *Os11g0696600* | Forward | CCTCCTCTTCATCGGATGGG |
|  | Reverse | GGTCCCCTTTCTTCTGCACA |
| *Os11g0703900* | Forward | GCAGGATTTCTTCAACGGCA |
|  | Reverse | TTGCCCTCACCACTCAAGAT |

**Supplementary Table S2.** QTL information for each trait identified in the Cheongcheong/Nagdong double haploid population.

| Characteristics | Year | QTL | Chromosome | Marker interval^z^ | LOD | Additive effect^y^ | *R^2^*^x^ | Increasing effects^w^ |
| --- | --- | --- | --- | --- | --- | --- | --- | --- |
| Winkler scale (°C) | 2018 | *qWS4* | 4 | RM551-RM1205 | 3.2 | -0.3 | 0.10 | Nagdong |
|  | 2018 | *qWS8* | 8 | RM1148-RM22334 | 3.7 | -0.2 | 0.13 | Nagdong |
|  | 2019 | *qWS8-1* | 8 | RM1148-RM22334 | 4.2 | -0.2 | 0.15 | Nagdong |
|  | 2020 | *qWS4-1* | 4 | RM551-RM1205 | 3.7 | -0.3 | 0.11 | Nagdong |
| Growth day (days) | 2018 | *qGD8* | 8 | RM6999-RM22334 | 3.8 | -0.3 | 0.08 | Nagdong |
|  | 2019 | *qGD8-1* | 8 | RM6999-RM22334 | 3.8 | -0.1 | 0.10 | Nagdong |
|  | 2020 | *qGD4* | 4 | RM551-RM1205 | 3.3 | -0.1 | 0.10 | Nagdong |
| Heading date (days) | 2018 | *qHD8* | 8 | RM6999-RM22334 | 3.1 | -0.3 | 0.08 | Nagdong |
|  | 2019 | *qHD8-1* | 8 | RM6999-RM22334 | 3.3 | -0.2 | 0.09 | Nagdong |
|  | 2019 | *qHD9* | 9 | RM3769-RM444 | 4.4 | -0.1 | 0.13 | Nagdong |
|  | 2020 | *qHD4* | 4 | RM551-RM1205 | 3.3 | -0.3 | 0.10 | Nagdong |
| Amylose content (%) | 2018 | *qAC8* | 8 | RM6999-RM22334 | 4.5 | -0.3 | 0.14 | Nagdong |
|  | 2018 | *qAC11* | 11 | RM3428-RM26771 | 3.1 | -0.4 | 0.10 | Nagdong |
|  | 2019 | *qAC8-1* | 8 | RM6999-RM22334 | 4.5 | -0.3 | 0.14 | Nagdong |
|  | 2019 | *qAC11-1* | 11 | RM3428-RM26771 | 3.1 | -0.4 | 0.10 | Cheongcheong |
|  | 2020 | *qAC8-2* | 8 | RM6999-RM22334 | 3.9 | -0.3 | 0.12 | Nagdong |
|  | 2020 | *qAC2* | 2 | RM3512-RM6 | 3.4 | -0.3 | 0.12 | Nagdong |
| Yield (kg/10a) | 2018 | *qYD7* | 7 | RM8261-RM420 | 3.1 | -0.3 | 0.11 | Cheongcheong |
|  | 2018 | *qYD3* | 3 | RM218-RM7197 | 3.8 | -0.2 | 0.12 | Cheongcheong |
|  | 2019 | *qYD6* | 6 | RM3343-RM439 | 3.6 | -0.3 | 0.12 | Cheongcheong |
|  | 2019 | *qYD8* | 8 | RM6999-RM22334 | 3.7 | -0.3 | 0.08 | Nagdong |

^z^ Interval markers are those within the significance threshold on each border of the QTL range

^y^ Positive values of the additive effect indicate that alleles from Cheongcheong are in the direction of increasing the traits

^x^ The proportion of evaluated phenotype variation attributable to a particular QTL was estimated by the coefficient of determination (*R^2^*)

^w^ Increase allele is the source of the allele causing an increase in the measured trait

**Supplementary Table S3.** Information of potential candidate genes against to winkler scale in rice.

| Chromosome | Marker interval | Locus | Description |
| --- | --- | --- | --- |
| 2 | RM3512-RM6 | *Os02g0758000* | Similar to Low molecular weight heat shock protein precursor |
|  |  | *Os02g0760000* | Heat shock protein DnaJ family protein |
|  |  | *Os02g0774300* | Similar to Heat shock 70 kDa protein, mitochondrial precursor |
|  |  | *Os02g0766700* | Similar to Abscisic acid responsive elements-binding factor |
|  |  | *Os02g0769100* | Auxin responsive SAUR protein family protein |
|  |  | *Os02g0771700* | Glycoside hydrolase, family 17 protein |
|  |  | *Os02g0774500* | Similar to DNA-3-methyladenine glycosylase |
|  |  | *Os02g0785000* | Glycosyl transferase, family 31 protein |
|  |  | *Os02g0790500* | Glycosyl transferase, family 20 domain containing protein |
|  |  | *Os02g0765300* | Similar to Alpha-amylase precursor |
|  |  | *Os02g0765600* | Alpha-amylase precursor |
|  |  | *Os02g0791400* | Cytochrome oxidase c, subunit VIb family protein |
| 3 | RM218-RM7197 | *Os03g0218500* | Similar to 70kD heat shock protein |
|  |  | *Os03g0255200* | Heat shock protein DnaJ, N-terminal domain containing protein |
|  |  | *Os03g0276800* | Heat shock protein Hsp70 family protein |
|  |  | *Os03g0245800* | Similar to Heat shock protein 26 |
|  |  | *Os03g0180700* | Glycosyl transferase, group 1 domain containing protein |
|  |  | *Os03g0184300* | Glycosyl transferase, family 8 protein |
|  |  | *Os03g0211700* | Glycoside hydrolase family 79, N-terminal protein |
|  |  | *Os03g0211800* | Glycosyl transferase, family 8 protein |
|  |  | *Os03g0276900* | Glycosyl transferase, family 14 protein |
|  |  | *Os03g0223100* | Cytochrome P450 family protein |
|  |  | *Os03g0168600* | Cytochrome P450 family protein |
|  |  | *Os03g0251000* | Plant lipid storage/trypsin-alpha amylase inhibitor domain containing protein |
| 4 | RM551-RM1205 | *Os04g0415900* | Plant lipid storage/trypsin-alpha amylase inhibitor domain containing protein |
|  |  | *Os04g0462200* | Plant lipid storage/trypsin-alpha amylase inhibitor domain containing protein |
|  |  | *Os04g0526600* | Similar to Alpha-amylase/subtilisin inhibitor |
|  |  | *Os04g0445100* | Similar to 22.7 kDa class IV heat shock protein precursor |
|  |  | *Os04g0538000* | Heat shock chaperonin-binding domain containing protein |
|  |  | *Os04g0549600* | Heat shock protein DnaJ family protein |
|  |  | *Os04g0568700* | Similar to Heat stress transcription factor Spl7 |
|  |  | *Os04g0457500* | Similar to Gamma-glutamyltranspeptidase 1 precursor |
|  |  | *Os04g0490600* | Nucleotide-sugar transporter family protein |
|  |  | *Os04g0624600* | Similar to Starch synthase DULL1 |
| 6 | RM3343-RM439 | *Os06g0613600* | Cytochrome P450 family protein |
|  |  | *Os06g0639800* | Cytochrome P450 family protein |
|  |  | *Os06g0650900* | Heat shock protein DnaJ family protein |
|  |  | *Os06g0679800* | Heat shock protein Hsp70 family protein |
|  |  | *Os06g0711700* | Galactose oxidase, central domain containing protein |
|  |  | *Os06g0726400* | Branching enzyme-I precursor (Starch-branching enzyme I) |
|  |  | *Os06g0643800* | Similar to Sucrose-phosphate synthase 7 |
|  |  | *Os06g0687900* | Glycosyl transferase, family 43 protein |
|  |  | *Os06g0702600* | Similar to Auxin response factor 7a |
| 7 | RM8261-RM420 | *Os07g0620200* | Heat shock protein DnaJ, N-terminal domain containing protein |
|  |  | *Os07g0632600* | Heat shock protein DnaJ, N-terminal domain containing protein |
|  |  | *Os07g0637100* | Heat shock protein DnaJ, N-terminal domain containing protein |
|  |  | *Os07g0689800* | Heat shock protein DnaJ, N-terminal domain containing protein |
|  |  | *Os07g0635200* | Cytochrome P450 family protein |
|  |  | *Os07g0647200* | Cytochrome P450 family protein |
|  |  | *Os07g0622300* | UDP-glucuronosyl/UDP-glucosyltransferase family protein |
|  |  | *Os07g0627000* | Glycoside hydrolase, family 77 protein |
|  |  | *Os07g0661100* | Glycosyl transferase, family 4 protein |
|  |  | *Os07g0616800* | Sucrose synthase 3 |
| 8 | RM6999-RM22334 | *Os08g0127600* | Heat shock protein DnaJ, N-terminal domain containing protein |
|  |  | *Os08g0323600* | Glycoside hydrolase, starch-binding domain containing protein |
|  |  | *Os08g0359500* | HSP20-like chaperone domain containing protein |
|  |  | *Os08g0381600* | Similar to Alpha-amylase/trypsin inhibitor (Antifungal protein) |
|  |  | *Os08g0464000* | Activator of Hsp90 ATPase homologue 1-like family protein |
|  |  | *Os08g0473600* | Alpha-amylase isozyme 3E precursor |
|  |  | *Os08g0473900* | Alpha-amylase isozyme 3D precursor |
|  |  | *Os08g0474600* | Heat shock protein DnaJ, N-terminal domain containing protein |
|  |  | *Os08g0477700* | Heat shock protein DnaJ, N-terminal domain containing protein |
|  |  | *Os08g0500700* | Similar to Heat shock protein 82 |
|  |  | *Os08g0532800* | Plant lipid transfer/trypsin-alpha amylase inhibitor domain containing protein |
|  |  | *Os08g0546800* | Similar to Heat shock transcription factor 33 |
| 9 | RM3769-RM444 | *Os09g0460000* | Heat shock protein DnaJ, N-terminal domain containing protein |
|  |  | *Os09g0482400* | Similar to Heat shock protein 82 |
|  |  | *Os09g0482600* | Heat shock protein 82 |
|  |  | *Os09g0482900* | UDP-glucuronosyl/UDP-glucosyltransferase family protein |
|  |  | *Os09g0490400* | Glycoside hydrolase, family 1 protein |
|  |  | *Os09g0469400* | Similar to Isoamylase-type starch debranching enzyme ISO3 |
|  |  | *Os09g0504000* | Similar to Nucleotide sugar epimerase-like protein |
| 11 | RM3428-RM26771 | *Os11g0187500* | Similar to Heat shock protein 70 |
|  |  | *Os11g0187600* | Similar to Heat shock protein 70 |
|  |  | *Os11g0244200* | Similar to Pisum sativum 17.9 kDa heat shock protein |
|  |  | *Os11g0578100* | Heat shock protein DnaJ family protein |
|  |  | *Os11g0578500* | Heat shock protein DnaJ family protein |
|  |  | *Os11g0696600* | Heat shock protein Hsp70 family protein |
|  |  | *Os11g0703900* | Heat shock protein 70 |

**
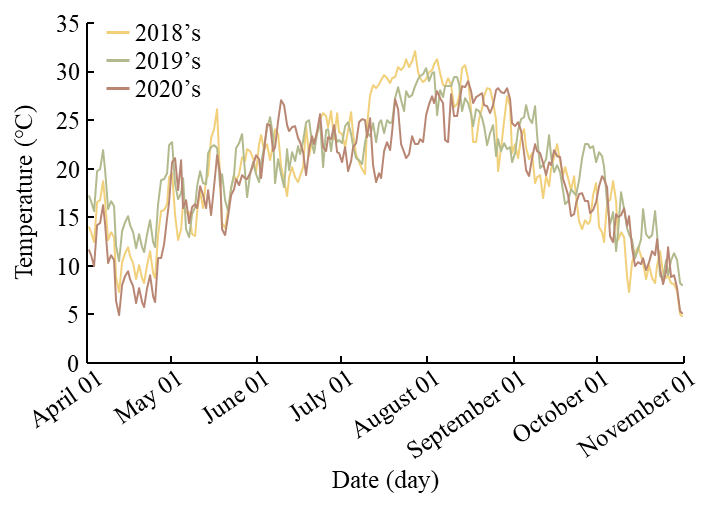
**

**Supplementary Figure S1. Average daily temperature in the field during rice growth.** Average daily temperature measured in rice fields of Kyungpook National University for three consecutive years from 2018 to 2020. The highest temperatures in 2018, 2019, and 2020 are 32.1°C, 30.4°C, and 29.0°C, respectively. The lowest temperature during the survey period was 4.4 °C in 2018, 8.0 °C in 2019, and 4.9 °C in 2020.

**
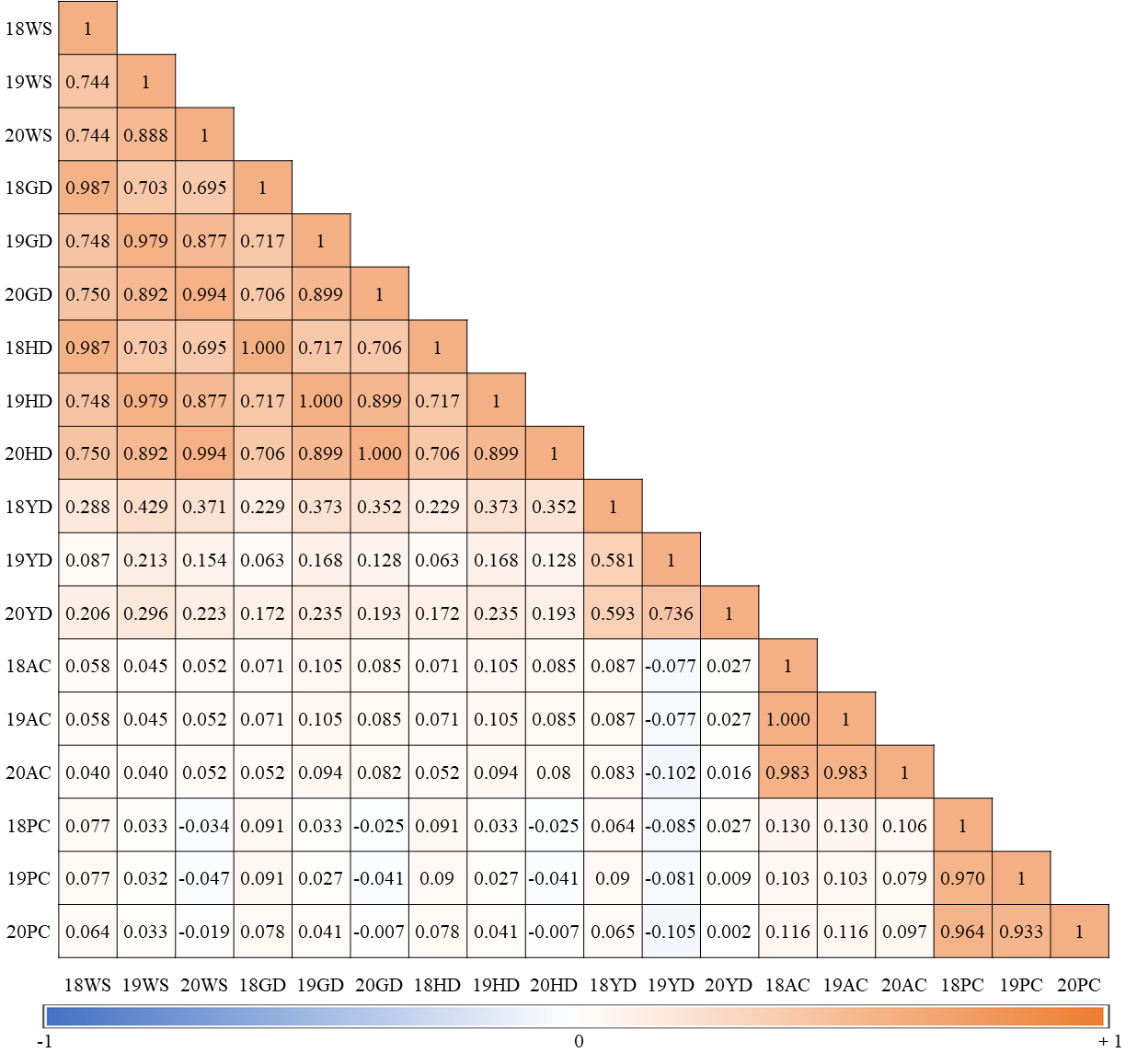
**

**Supplementary Figure S2. Correlations of agronomic traits with temperature during the growing season in each year.** Each sector represents positive or negative values for correlation. The darker the color for the sector, the larger the absolute value. The number in the box is the correlation coefficient for each trait. A change from dark orange to dark blue corresponds to a change in the correlation coefficient from +1 to -1. For the same trait, it was maintained at a similar level with no significant difference by year. WS, winkler scale; GD, growth day; HD, heading date; YD, yield; AC, amylose content; PC, protein content.


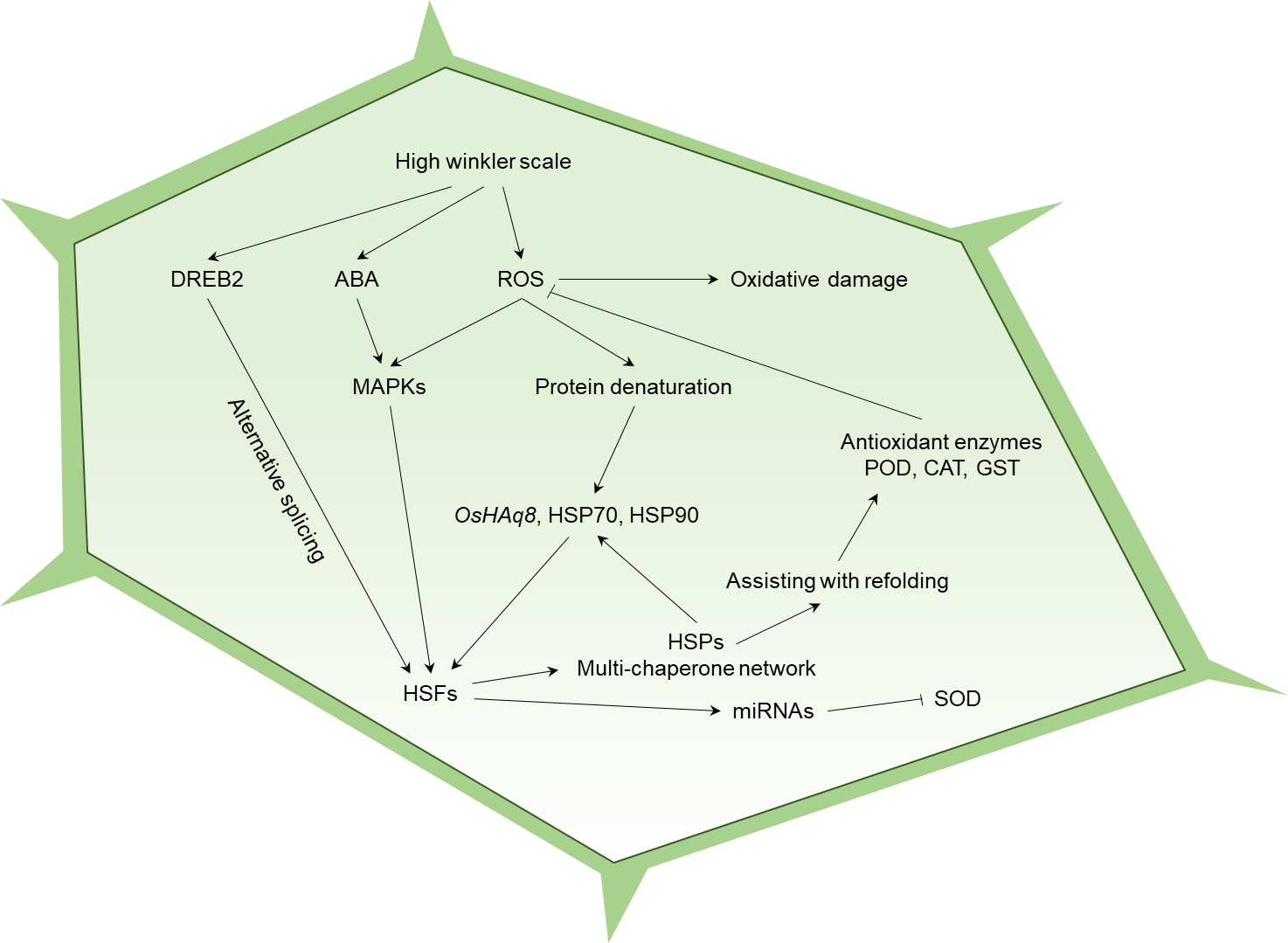


**Supplementary Figure S3**. **Interaction scheme to correspond to the Winkler scale of *OsHAq8*.** *OsHAq8* and heat shock proteins assist protein refolding to remove stress-induced ROS and prevent protein denaturation. ROS (Reactive oxygen species), ABA (Abscisic acid), MAPK (Mitogen-activated Protein Kinase), DREB (Dehydration responsive element binding protein), POD (Peroxidase), CAT (Catalase), SOD (Superoxide dismutase).
